# The impact of COVID-19 on a cohort of patients treated with clozapine

**DOI:** 10.1017/ipm.2021.30

**Published:** 2021-04-05

**Authors:** Y. Fahy, B. Dineen, C. McDonald, B. Hallahan

**Affiliations:** 1 Department of Medicine, Galway University Hospital, Galway, Ireland; 2 School of Medicine, National University of Ireland Galway, Galway, Ireland; 3 Galway–Roscommon Mental Health Services, University Hospital Galway, Ireland

**Keywords:** Anxiety, COVID-19, clozapine, schizophrenia, social interaction

## Abstract

**Objectives:**

To examine the psychological and social impact of the COVID-19 pandemic and its associated restrictions on a cohort of patients with severe and enduring mental illness treated with clozapine.

**Methods:**

Semi-structured interviews were conducted with 63 individuals attending a clozapine clinic within the Galway–Roscommon Mental Health Services to determine the impact of COVID-19 restrictions on anxiety and depressive symptoms, social and occupational functioning and quality of life, by utilising Likert scale data. The Beck Anxiety Inventory (BAI) and Hamilton Anxiety Rating Scale (HAM-A) were additionally utilised to measure anxiety symptoms cross-sectionally.

**Results:**

Anxiety symptoms were low with a median BAI score of 4.0 and HAM-A score of 4.0. Likert scale measurements recorded only a modest adverse impact of COVID-19 restrictions on anxiety and depressive symptoms, quality of life and occupational and social functioning. Free-text comments from patients (*n* = 55), were grouped into five themes: neutral impact (*n* = 22), negative psychological impact (*n* = 13), negative social impact (*n* = 11), positive psychological impact (*n* = 5) and media coverage inducing anxiety (*n* = 4).

**Conclusions:**

Three months into the COVID-19 pandemic and its restrictions, the impact on individuals with treatment-resistant psychotic disorders attending a clozapine clinic has been modest, with preliminary evidence demonstrating minimal increases in subjective symptoms of anxiety and reduced social functioning. Reduced social engagements and supports attainable both within the community and from mental health services were noted by some participants.

## Introduction

COVID-19 is the infectious disease associated with the recently discovered coronavirus, SARS-CoV-2. First identified in Wuhan, China, in December 2019, COVID-19 was characterised as a pandemic by the World Health Organization (WHO) on 11 March 2020 (World Health Organisation, 2020). The COVID-19 pandemic has dominated media coverage in recent months with updates regarding spread and lethality produced and available on a minute-by-minute basis (González-Padilla & Tortolero-Blanco, 2020). The declaration of the pandemic was followed by the implementation of restrictions and ‘“lockdowns’ in many countries worldwide. In Ireland, restrictions included ‘cocooning’ of individuals over 70 years of age, a limitation on travel and the introduction of social distancing measures, which resulted in the closure of many facilities deemed as ‘non-essential’. In addition to restaurants and cafes, these facilities included centres attended by individuals with mental health disorders such as day centres and training centres where group activities and social engagement occurred. Whilst the potentially devastating medical, economic and cultural impacts of a viral pandemic are well established (Nicola et al., 2020), the social impact can also be very significant. Social effects of a pandemic may include disruption of daily routine and social isolation including separation from family and friends (Taylor, 2019).

Increasing debate and discussion in medical literature and in social media has surrounded the potential adverse psychological or psychiatric sequelae relating to COVID-19. Previous viral pandemics have been associated with increased psychological distress (WHO ‘Outbreak Communication Guidelines’, 2005), with perspective pieces (Kelly, 2020) and some initial research studies noting an increase in psychiatric pathology, including higher levels of depressive and anxiety symptoms, in individuals with no prior mental disorder subsequent to mandated government restrictions secondary to COVID-19 (Wang et al., 2020). However, some limited data in individuals attending mental health services for the management of pre-existing anxiety disorders have noted a relatively modest impact of the COVID-19 pandemic on psychopathology or social well-being (Plunkett et al., 2020).

To our knowledge, there have been no published studies to date examining the impact of the COVID-19 pandemic in relation to anxiety symptoms and social impact for individuals with pre-existing diagnosed treatment-resistant schizophrenia or other treatment-resistant psychotic disorders. However, a number of studies conducted have suggested that individuals with schizophrenia or other mental disorders may be at risk of increased symptomatology related to the COVID-19 pandemic. These studies include a case register study of individuals with mental disorders including schizophrenia with ‘pandemic related psychiatric symptoms’ including anxiety symptoms documented in approximately 12% of patients (Rohde et al., 2020) and a study of individuals with a range of mental health disorders including schizophrenia, who had modestly increased levels of distress and anxiety symptoms compared to individuals with no prior history of a mental health disorder (Iasevoli et al., 2020). Additionally, individuals with a diagnosis of schizophrenia admitted to the hospital with suspected COVID-19 have been noted to experience increased levels of anxiety symptoms and distress compared to those admitted to the hospital for other medical illnesses mental health disorder (Liu et al., 2020).

The reduced availability of many communities and mental health supports such as day centres and training centres may potentially place this patient cohort at an increased risk of adverse psychological sequelae secondary to the COVID-19 pandemic. This patient cohort is also potentially at greater risk due to often higher rates of isolation and loneliness compared to the general population (O’Connor *et al.*, 2020). In this study, we aimed to assess the psychological and social impact of COVID-19 including its associated mandated social restrictions on this cohort of individuals treated with clozapine with treatment-resistant psychotic disorders. We hypothesised that patients would have experienced increased symptoms of anxiety and impaired social functioning.

## Materials and methods

### Participants

All patients were invited to participate in this study and attended a dedicated clozapine clinic at the Galway University Hospital for mandatory full blood count and physical health monitoring and provision of clozapine medication, and all have a treatment-resistant psychotic disorder. Inclusion criteria for the study required patients to be on clozapine treatment, who are over 18 years of age and have the capacity to provide written informed consent for study participation. Participants were excluded if they fulfilled the criteria for an intellectual disability (intelligence quotient <70) or had a confirmed diagnosis of dementia. Following application of inclusion and exclusion criteria (*n* = 35, due to impaired capacity to participate), 142 participants were invited for study participation. Research interviews were undertaken by one author (YF) with training in study procedures provided by the principal investigator (BH). All responses were anonymised and all data were stored securely and handled in accordance with the Data Protection Act, 2018. Ethical approval was attained prior to study commencement from the Galway University Hospitals Research Ethics Committee (C.A. 1462).

### Procedure

For individuals providing written informed consent (*n* = 63, 44.4%), clinical case notes were reviewed to attain basic demographic and clinical data. Demographic data included age, gender, marital, domiciliary and employment or vocational status. Clinical data included psychiatric diagnosis, dose of clozapine, duration of clozapine treatment, comorbid mental health disorder, physical illness and other prescribed psychotropic medications including first- or second-generation anti-psychotic medication, alcohol, tobacco and psychoactive substance use.

### Assessments

A semi-structured interview was conducted either in person (*n* = 48) or by telephone (*n* = 15) with participants by the same researcher (YF), who provided written informed consent between 24 June and 21 August 2020, approximately 15–23 weeks after government-mandated social restrictions (referred to anecdotally as ‘lockdown’) had commenced. At this time, some initial restrictions were in the process of being eased (i.e. restaurants were allowed to reopen with restrictions on customer numbers on 29 June 2020).

Demographic and clinical variable data (see Table 1.) attained from clinical note review was supplemented where required by data attained from clinical interviews, with additional information pertaining to physical health, current domiciliary status and effect of COVID-19 on the participants’ employment or vocational status or site of employment collected.

Participants’ subjective experience of the impact of COVID-19 pandemic on their mental health status was measured utilising a study-specific Likert scale (0–10) to measure: (1) anxiety symptoms, (2) mood symptoms, (3) social functioning, (4) occupational functioning and (5) quality of life; with 0 indicating no adverse impact and 10 indicating a very severe impact due to restrictions imposed because of the COVID-19 pandemic (see Appendix 1 for Likert scale and instructions and supports for participants in completing these scales).

Established psychometric instruments with known high reliability and validity indices were also utilised to measure current symptomatology: (1) Beck Anxiety Inventory (BAI, Beck & Steer, 1993) and (2) Hamilton Anxiety Rating Scale (HAM-A, Hamilton 1959). The BAI and HARS have previously been utilised and validated for use in individuals with psychotic disorders (Smith et al., 2019, Soraya et al., 2007). The rationale for using two psychometric instruments (BAI and HAM-A) was to measure both subjective and objective symptoms of anxiety, increasing the accuracy of recording of anxiety symptoms with the aim of reducing any scale bias and to increase confidence in findings pertaining to anxiety symptoms in this patient cohort. A bivariate correlation analysis was conducted between the Likert scale variables (anxiety, mood, social functioning, occupational functioning and quality of life) and these two validated psychometric scales.

Free-text data were additionally collected and enabled participants to clarify the impact the COVID-19 pandemic and its associated mandated restrictions had on their mental state and overall functioning.

### Statistical analysis

Statistical analysis was performed using the Statistical Package for Social Sciences (SPSS) 24.0 for Windows (SPSS Inc., IBM, USA). Descriptive analyses (frequencies, percentages, means and standard deviation) on key demographic and clinical data were performed for both categorical and continuous variables as appropriate. Where data were not normally distributed, median values and interquartile ranges (IQRs) were attained. Data were examined to determine if normally distributed by visual inspection utilising histograms and by Q–Q plots and non-parametric testing of continuous data utilising the Mann–Whitney U test were additionally undertaken as appropriate. All statistical tests were two-sided and the α-level for statistical significance was 0.05. Correlation of Likert scale data with other Likert scale variables and with the BAI and HARS was performed utilising non-parametric Spearman’s rho correlation (*ρ*). Free-text data were examined and were open-coded based on the framework of the questionnaire and on any other themes unrelated to these questions that emerged. This data attained from free texts was then grouped into themes by consensus of the researchers (YF, CMcD, BH).

## Results

### Demographics and clinical data

A total of 142 participants were initially invited to participate, of which 20 were uncontactable (i.e. not answering phone calls, attending their local GP rather than the clozapine clinic during COVID-19) and 59 refused to participate, resulting in a 44.4% response rate. There was no significant difference in terms of gender, age or diagnosis between respondents and non-respondents. Data for the 63 study participants is presented in Table 1. Of note, 44 (69.8%) participants were male, the mean age of participants was 44.7 ± 10.6 years, 23 participants were engaged in employment prior to ‘lockdown”, with 5 having their site of employment temporarily terminated and 4 of the remaining 18 participants having their site of work moved to their own residence due to COVID-19 restrictions. Fifty-nine (93.7%) participants had a diagnosis of schizophrenia and the mean duration of clozapine treatment was 11.6 ± 6.9 years. Eight individuals had a comorbid mental health disorder, most commonly anxiety disorder (*n* = 7, 11.1%).

**Table 1.** Demographics and clinical data

Variables	*n* (%)
**Gender**	
Male	44 (69.8)
Female	19 (30.2)
**Marital status**	
Single	49 (77.8)
Married/civil partnership	10 (15.9)
Separated/divorced	4 (6.3)
**Employment**	
Unemployment	35 (55.6)
Employed	18 (28.6)
Lost employment (COVID-19)	5 (7.9)
In third level education/attending a course	5 (7.9)
**Domiciliary status**	
Parents	24 (38.1)
Partner/spouse	9 (14.3)
Other family members	4 (6.3)
Housemates/friends	0 (0.0)
Group home	11 (17.5)
Alone	15 (23.8)
**Primary diagnosis**	
Schizophrenia	56 (88.9)
Schizoaffective disorder	5 (7.9)
Other disorders^a^	2 (3.2)
**Comorbid psychiatric disorder**	
Anxiety disorders^b^	7 (11.1)
Major depressive disorder	1 (1.6)
**Substance use**	
Alcohol	15 (23.8)
Nicotine	26 (41.3)
Cannabis	0 (0.0)
Other psychoactive substances	0 (0.0)
**Comorbid physical disorder**	
Diabetes mellitus	3 (4.8)
COAD/ssthma	1 (1.6)
Other physical disorders^c^	8 (12.7)
**Other anti-psychotic medication**	
First-generation anti-psychotic (FGA)	12 (19.0)
Second-generation anti-psychotic (SGA)	3 (4.8)
	**Mean (SD)**
**Age**	44.7 (10.6)
**Clozapine**	
Duration of clozapine treatment (years)	11.6 (6.9)
Current dose of clozapine (mg)	317.3 (132.1)

COAD, Chronic Obstructive Airway Disease.

a Includes Parkinson’s disease with psychosis, intellectual disability with psychosis.

b Includes Obsessive-Compulsive Disorder (OCD) (*n* = 3).

c Includes neurological and musculoskeletal disorders.

### Clinical effects of COVID-19

Low levels of anxiety symptoms were demonstrated as measured subjectively utilising the BAI (median = 4.0, IQR = 10.3) [the maximum score on this scale is 63 and under 7 is considered minimal] and objectively utilising the HAM-A (median 4.0, IQR 9.0) [the maximum score on this scale is 56 and under 7 is considered minimal]. Likert scale data demonstrated a minor impact over time of COVID-19 on symptomatology (anxiety or mood symptoms), quality of life, social functioning (Table 2) with the least impact noted for occupational functioning (median = 0, IQR = 5.0). When clinical variables were examined, only increasing age was associated with increased anxiety symptoms on the Likert scale (*ρ* = 0.33, *p* = 0.008). No significant association was demonstrated between increasing anxiety symptoms and a range of clinical variables including having a comorbid mental health disorder (*U* = 232, *p* = 0.105), physical health disorder (*U* = 263, *ρ* = 0.664) or participants’ employment status (*U* = 350, *p* = 0.389). Likert scale data yielded moderate correlations with psychometric instruments, with anxiety symptoms correlating and with the BAI and HAM-A at a moderate level (*ρ* = 0.56, *p* < 0.001) (see Appendix 2).

**Table 2.** Symptoms displayed

Impact of COVID-19: Likert Scale Data	Median	IQR
Anxiety symptoms	3.0	6.0
Mood symptoms	2.0	5.0
Functioning: social	3.0	6.0
Functioning: occupational	0.0	5.0
Quality of life	3.0	5.0
**Anxiety symptoms**		
BAI	4.0	10.3
HAM-A	4.0	9.0

BAI, Beck Anxiety Inventory; HAM-A, Hamilton Anxiety Rating Scale; IQR, Interquartile range.

Anxiety symptoms are correlated with BAI and HAM-A scales (*ρ* = 0.56, *p* < 0.001 for both).

Data were not normally distributed.

### Qualitative data

Fifty-five (87.3%) participants provided free-text responses when asked if they would like to provide additional comments regarding their thoughts or feelings pertaining to the COVID-19 pandemic. In total, five themes emerged: (1) neutral effects of COVID-19 (*n* = 22), (2) negative psychological impact of COVID-19 (*n* = 13), (3) negative social impact of COVID-19 (*n* = 11), (4) psychological benefits of COVID-19) (*n* = 5), (5) media coverage of COVID-19 inducing anxiety (*n* = 4)) (Box 1). These comments highlighted that whilst many participants had no adverse sequelae secondary to the COVID-19 pandemic, the reduction in social contact, often organised or associated with mental health or other health services was a significant concern and/or a source of distress for some patients. Additionally, several patients reported increased anxiety or an increase in subjective low mood secondary to isolation or excessive engagement with media coverage of the COVID-19 pandemic.

Box 1. Themes emanating from free-text responses: Patient comments regarding their experiences during the COVID-19 pandemic

Theme 1: Neutral effects of COVID-19 (*n* = 22)

‘COVID-19 hasn’t affected me very much; I just kept going the same way as I always have been’. (#51, female)

‘Didn’t bother me at all’. (#39, male)

‘It made no difference to me; I didn’t go out beforehand anyways’. (#54, male)

‘Because I already have a mental illness, I felt immune to the symptoms people were experiencing during lockdown because I am used to being in a routine. I live in a bubble, my own little lockdown all the time. I am good at making my own structure for my day’. (#43, male)

Theme 2: Negative psychological impact of COVID-19 (*n* = 13)

‘It made me very anxious, so hard not being allowed to go home and see my family’. (#13, female)

‘I felt isolated and unhappy; I couldn’t even see my brother’. (#15, male)

‘I was having more anxiety attacks, felt really nervous about spreading or getting COVID-19’. (#17, male)

Theme 3: Negative social impact of COVID-19 (*n* = 11)

‘I really missed the training centre; I had a lot of time on my hands to think’. (#29, male)

‘I was disappointed that my language classes were cancelled. I preferred the social side of classes compared to the online classes’. (#45, male)

‘I found it very hard when my AA meetings were stopped’. (#55, male)

Theme 4: Psychological benefits of COVID-19 (*n* = 5)

‘I felt more calm and relaxed’. (#63, male)

‘It slowed everything down; I found it good’. (#28, female)

‘There was more peace, I enjoyed it’. (#49, male)

Theme 5: Media coverage of COVID-19 inducing anxiety (*n* = 4)

‘I feel panicked listening to the news’. (#56, female)

‘Too many updates all the time on the radio and TV, it made it harder’. (#12, male)

‘I worried hearing constant updates’. (#47, male)

Fifty-five participants were provided with free-text comments.

## Discussion

To our knowledge, this is the first study to date to examine the impact of COVID-19 and its mandated restrictions for individuals with treatment-resistant psychotic disorders, who are attending secondary mental health services for clozapine treatment. A number of studies to date have suggested that individuals with schizophrenia or other mental disorders may be at increased risk of symptomatology in relation to COVID-19. For example, Rohde et al., 2020 reported ‘pandemic related psychiatric symptoms” in approximately 12% of patients with mental disorders, Laveoli et al., 2020 noted increased depressive symptomatology and distress in individuals with mental health disorders including schizophrenia and Liu et al., 2020 noted that individuals with schizophrenia displayed greater levels of anxiety symptoms if they were being treated for probable COVID-19 compared to other physical health difficulties. This study consequently expands on this current literature base, assessing anxiety symptoms utilising both psychometric instruments and Likert scale measurements and additionally including measures pertaining to functioning and quality of life. Participants reported some negative impact of COVID-19 on anxiety symptoms; however, this impact was not marked. Participants also noted an impact of the COVID-19 pandemic on social functioning, with this impact most evident from qualitative analysis. Increasing age was associated with higher levels of worsening anxiety amongst this patient cohort, but no other clinical factors were associated with increased anxiety or distress secondary to the COVID-19 pandemic.

To date, media reports, often based on existing data from previous pandemics, combined with emerging data from this pandemic, and perhaps anecdotal evidence have suggested that psychological morbidity will inevitably rise (Maunder et al., 2008), with several suggestions of an upcoming ‘tsunami of mental health difficulties’ or ‘mental health crisis’ as a consequence of the COVID-19 pandemic (Pedrosa et al., 2020). However, consistent with a recent study on individuals attending secondary mental health services with pre-existing anxiety disorders (Plunkett *et al.*, 2020); we demonstrated only a modest impact from the first wave of the COVID-19 pandemic on individuals’ psychopathology. Of note, some patients did report, by utilising Likert scales an increase in anxiety symptoms, reinforced by the correlation of this item with the validated cross-sectional assessment of anxiety using psychometric scales. Free-text comments suggest that anxiety symptoms were associated with increased social isolation, including less available supports or contacts from family supports, in the community and from mental health services (i.e. day centres or training centres). Indeed, this patient cohort is likely at greater risk of anxiety secondary to reduced social supports, given higher rates of isolation and loneliness compared to the general population for individuals with schizophrenia (O’Connor et al., 2020). This study highlights the importance of prioritising community and group activities for this patient cohort, whilst adhering to social distancing and government guidance.

Consistent with previous data, overconsumption of media regarding reports of infectious diseases can result in increased anxiety symptoms (Holmes et al., 2020). Media coverage has increased anxiety symptoms for some individuals in this patient cohort. Potentially, if some media coverage was re-framed to encourage and promote coping strategies and health-sustaining behaviours (Basch, et al., 2020), the deleterious sequelae secondary to the current media portrayal of COVID-19 could be reduced.

There are a number of putative reasons why individuals with treatment-resistant psychotic disorders (treated with clozapine) in this study have, despite 4 months of restrictions in an active viral pandemic, not demonstrated a significant deterioration in symptomatology. First, this cohort of patients, despite the presence of enduring mental disorder are predominantly stable from a mental health perspective, and despite the limited availability of some community and mental health supports continue to attain input from their mental health team and a dedicated clozapine clinic. Second, face-to-face contact is required for this patient cohort, given the mandated need for full blood count monitoring and dispensing of medication. Thus, this cohort unlike other patient cohorts not treated with clozapine are likely attaining greater levels of support from the mental health services including more face-to-face contact. Third, perhaps unlike individuals without a pre-established mental disorder or individuals experiencing anxiety symptoms or other symptoms of mental disorder *de novo*, participants have an awareness of how to access supports and are aware of techniques to reduce their distress due to their engagement with mental health services. Fourth, despite reductions for some participants in social supports, many individuals with enduring mental health disorders such as schizophrenia have a relatively narrow repertoire of activities and as noted particularly in the qualitative comments section, restrictions secondary to the COVID-19 pandemic have to date not significantly impacted their routine, or occupational or social life. Fifth, none of the individuals in this cohort engaged in harmful use of psychoactive substances and although 23.8% of the cohort consumed alcohol, no participant was engaged in consuming alcohol above the Health Service Executive recommended low-risk alcohol guidelines (less than 11 standard drinks for women and less than 17 standard drinks for men). Finally, a diagnosis of a mental disorder including a treatment-resistant psychotic disorder does not mitigate against an individuals’ ability to be resilient. It is likely in this cohort that many participants are engaged not alone in appropriate coping mechanisms, but are also demonstrating significant resilience (‘positive adaptation, or the ability to maintain or regain mental health, despite experiencing adversity’; Hermann et al, 2011).

There are a number of limitations to this study. First, only 44% of all individuals with the capacity to engage (and 36% of all individuals attaining clozapine treatment) at a specialised clozapine clinic participated in this study. Of those patients that were contactable, 52% agreed to participate, which is similar (*albeit* a little lower) than was noted in a previous study in the same cohort of patients (61%; Maher *et al.*, 2016). Concerns about spending time potentially viewed as not associated with their care may be a reason for this relatively low response rate, *albeit* this does not necessarily mean that patients who did not engage had higher levels of anxiety. Thus, this cohort may not be representative of all individuals attaining clozapine treatment. Second, this study only included a cohort of patients treated with clozapine, which additionally may not be a representative of all individuals diagnosed with psychotic disorders. Furthermore, some previous studies have noted higher rates of co-morbid anxiety or depressive disorders in relation to cohorts of patients with treatment-resistant schizophrenia than noted in this study (Temmingh et al., 2015), potentially also impacting the generalisability of the study findings. Consequently, caution is required in the interpretation of findings for all individuals with schizophrenia; however, to date no other cross-sectional studies have been conducted in this patient cohort. This study can serve as a pilot study for future research with larger number of participants across a range of mental health disorders. Third, given the cross-sectional nature of this study, it is difficult to ascertain if symptom measurements secondary to the restrictions posed by the COVID-19 pandemic will remain at a modest level. Thus, further reviews of this cohort longitudinally including when different levels of restrictions are in place, or further waves prolong the withdrawal of supports, will be required to fully elucidate the impact of the COVID-19 pandemic on this vulnerable patient cohort in the medium-to-long term. Fourth, this study did not explore the impact of the COVID-19 pandemic on psychotic symptomatology. Without baseline psychometric measurements of psychotic symptoms (Positive and Negative Syndrome Scale or Brief Psychiatric Rating Scale), we believe that a different study design will best explore this important point and we plan to conduct a mirror image study exploring if patients have experienced a greater rate of psychotic relapse in a 12-month period after, compared to a 12-month period prior to the commencement of the COVID-19 pandemic. Finally, Likert scale data are not validated, and would not be expected to provide identical scores to an assessment on psychometric instruments, given that they are only one measure and are assessing the impact of COVID-19 on symptomatology rather than specific symptoms. However, the validity of our findings is tentatively suggested by a moderate correlation (and statistically significant finding) between anxiety symptoms secondary to the COVID-19 pandemic and scores on the BAI and HAM-A (see Appendix 2).

## Conclusion

**Fig. 1.**
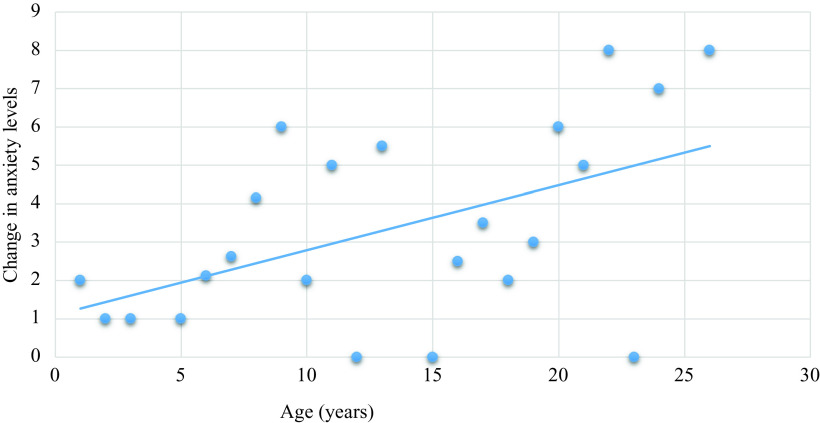
Change in anxiety levels associated with age.

Four months into the COVID-19 pandemic and its restrictions, the impact on individuals with treatment-resistant psychotic disorders attending a clozapine clinic has been modest, with preliminary evidence demonstrating minimal increases in subjective symptoms of anxiety and minimally reduced social functioning. Future research studies, including further more in-depth qualitative studies might more clearly delineate the potential adverse sequelae of more prolonged COVID-19 restrictions on individuals with diagnosed treatment-resistant psychotic disorders in the medium-to-long term and ascertain factors associated with positive and negative coping strategies during the COVID-19 pandemic. Such studies for this patient cohort will include mixed-method techniques to examine symptomatology and functioning longitudinally including after the COVID-19 pandemic resolves, which may enable exploration of how functioning during the COVID-19 pandemic impacts symptomatology and functioning when the COVID-19 pandemic resolves and social restrictions are no longer mandated. Additionally, it is aimed that this study will serve as a pilot study for future research with larger number of participants across a range of mental health disorders (Fig. 1).

## Appendix

**Appendix 1.** Likert Scale Data

Place a number in the box that best describes how the COVID-19 pandemic has affected you											
0 = No Effect.											
10 = Severe Effect.											
Anxiety levels	0	1	2	3	4	5	6	7	8	9	10
Mood Symptoms	0	1	2	3	4	5	6	7	8	9	10
Functioning: Social	0	1	2	3	4	5	6	7	8	9	10
Functioning: Occupation	0	1	2	3	4	5	6	7	8	9	10
Quality of Life	0	1	2	3	4	5	6	7	8	9	10
Additional comments: _________________________________________________________________________________________________________ _________________________________________________________________________________________________________ _________________________________________________________________________________________________________ _________________________________________________________________________________________________________											
Likert scale variables were explained to each individual thoroughly prior to questioning. Participants were informed that 0 equated to no impact, 1–3 equated to a mild impact, 4–6 equated to a moderate impact, 7–9 equated to a severe impact and 10 equated to a very severe impact. Participants were informed that the higher the number, the greater the impact of the COVID-19 pandemic for them and this was the same for all five items. Patients were advised that when scoring these items those scores had to relate to the impact COVID-19 had on their lives rather than their general mood/anxiety/functioning/quality of life. For example, in terms of social and occupational functioning and quality of life, patients were asked to briefly explain their typical routine prior to the COVID-19 pandemic and mandated restrictions and were subsequently asked to rate out of 10 how significantly these variables have been altered or impacted upon due to the COVID-19 pandemic, with 0 meaning no change and 10 being a very severe effect or impact. Each individual was given the opportunity to ask further questions to clarify the Likert scale ensuring their full understanding of the Likert scale.											

## Appendix

**Appendix 2.** Correlation of Likert Scale Data with Psychometric Instruments

	Anxiety	Mood	Social functioning	Occupational functioning	Quality of life	BAI	HARS
	*ρ*, *p*	*ρ*, *p*	*ρ*, *p*	*ρ*, *p*	*ρ*, *p*	*ρ*, *p*	*ρ*, *p*
**BAI**	0.56, <0.001	0.49, <0.001	0.50, <0.001	0.07, 0.60	0.44, <0.001	1.00, -	0.71, <0.001
**HAM-A**	0.56, <0.001	0.33, 0.015	0.41, 0.002	0.14, 0.30	0.33, 0.014	0.71, <0.001	1.00, -

BAI, Beck Anxiety Inventory, HAM-A, Hamilton Anxiety Rating Scale.
